# Ochre Bathing of the Bearded Vulture: A Bio-Mimetic Model for Early Humans towards Smell Prevention and Health

**DOI:** 10.3390/ani6010007

**Published:** 2016-01-15

**Authors:** Helmut Tributsch

**Affiliations:** Bio-Mimetics Program, Carinthia University for Applied Sciences, Europastrasse 4, Villach 9524, Austria; helmut.tributsch@alice.it

**Keywords:** *Gypaetus barbatus*, ochre, photo-sterilization, smell neutralization, disease prevention

## Abstract

**Simple Summary:**

The once widespread bearded vulture (*Gypaetus barbatus*) has the habit of bathing its polluted feathers and skin in red iron oxide-ochre-tainted water puddles. Primitive man may have tried to find out why: ochre is active in sunlight producing aggressive chemical species. They can kill viruses and bacteria and convert smelly organic substances into volatile neutral carbon dioxide gas. There is consequently a sanitary reason for the vulture’s habit of bathing in red ochre mud and this explains why prehistoric people included ochre use into their habits and rituals.

**Abstract:**

Since primordial times, vultures have been competing with man for animal carcasses. One of these vultures, the once widespread bearded vulture (*Gypaetus barbatus*), has the habit of bathing its polluted feathers and skin in red iron oxide - ochre - tainted water puddles. Why? Primitive man may have tried to find out and may have discovered its advantages. Red ochre, which has accompanied human rituals and everyday life for more than 100,000 years, is not just a simple red paint for decoration or a symbol for blood. As modern experiments demonstrate, it is active in sunlight producing aggressive chemical species. They can kill viruses and bacteria and convert smelly organic substances into volatile neutral carbon dioxide gas. In this way, ochre can in sunlight sterilize and clean the skin to provide health and comfort and make it scentless, a definitive advantage for nomadic meat hunters. This research thus also demonstrates a sanitary reason for the vulture’s habit of bathing in red ochre mud. Prehistoric people have therefore included ochre use into their rituals, especially into those in relation to birth and death. Significant ritual impulses during evolution of man may thus have developed bio-mimetically, inspired from the habits of a vulture. It is discussed how this health strategy could be developed to a modern standard helping to fight antibiotics-resistant bacteria in hospitals.

## 1. Introduction

### 1.1. The Attraction of Red Ochre for a Vulture

**Figure 1:**
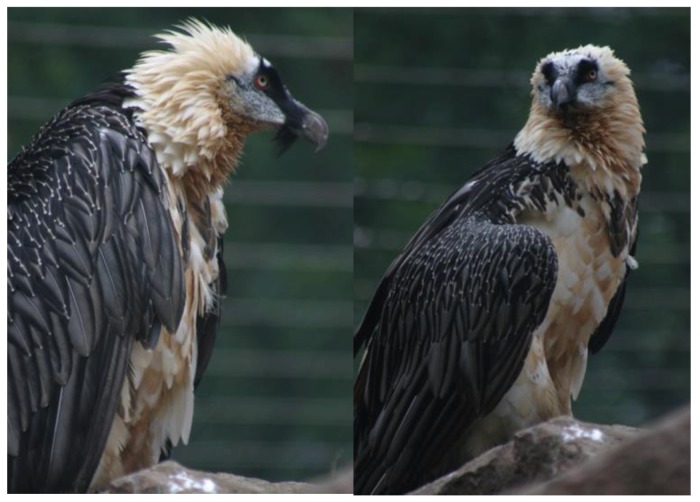
The bearded vulture (*Gypaetus barbaticus*) has a genetically anchored addiction to taint his feathers with red ochre mud.

Red muddy water has a spectacular effect on the bearded vulture (*Gypaetus barbatus*) (Figure 1). With wide opened eyes he approaches the mud and repeatedly stops to look at it. With closed eyes he then dips his head, shoulder and breast several times into the mud. Then the rest of the body follows until all white feathers are covered with mud. The red mud is then systematically rubbed into the feathers until they are reddish brown. Then the feathers are dried. This remarkable vulture is at present still roaming all over Eurasia, North Africa, and in sub-Saharan Africa from Sudan to Tanzania. In addition, it is found in southern Africa in localized regions like Lesotho, KwaZulu-Natal, and eastern Cape. Two subspecies are generally recognized. *Gypaetus barbatus meridionalis* occurs in east and southern Africa as well as in southwestern Arabia. *Gypaetus barbatus barbatus* occupies all the rest of its described range [1]. His preferred prey are bones of carcasses, which he drops to crack them from a height of up to 100 m. The vulture’s feeding behavior in Africa and in Europe respectively has been studied [2,3]. Also, his bone breaking behavior has been addressed in detail [4,5,6,7]. When this very dedicated carrion eating vulture with his wing span of up to 2.8 m was bred in captivity, it was observed, that he did not develop the conspicuous rust-red body plumage, seen in the wild. Not before 1995 did scientific studies demonstrate that in the free environment of their vast range they regularly search for iron oxide tainted water puddles in order to treat and taint their plumage. This habit is genetically fixed. When red mud is not available, as in captivity, the bearded vulture performs representative activities. In the Zoo of Basel a bearded vulture collected red autumn leaves in an effort to stain his feathers [8]. A photograph of a bearded vulture bathing in red ochre mud can be seen at this reference. In a documentary of animal life in Mongolia the bathing and tainting of the feathers of the bearded vulture has been filmed in the wild [9]. Scientifically, the coloration of the bearded vulture has received detailed attention in papers by Negro *et al.* [10,11] and other researchers [12,13]. The ochre bathing habit of the vulture has been analyzed both in terms of cosmetic and medical significance. The relevance of iron oxide as antibacterial agent has been investigated in some detail. However, the idea was essentially discarded, because of missing chemical and biochemical evidence for such activity and because iron is an essential component of bacterial function [10]. These authors, however, only considered chemical mechanisms of iron oxide proceeding in the dark. They were not aware that antibacterial activity based on iron oxide needs energy, solar light, to proceed. Their conclusions therefore do not interfere with the work presented here, in which the photochemical aspects of ochre activity are taken into consideration. Ochre bathing, as a strategy against feather wear, has also been discussed [14] but could not be confirmed [15]. Another theory, which was advanced, was relevance of ochre bathing in context with reproduction [12]. A counter argument is that juvenile vultures also do that several years before reproduction. A presently actively discussed theory is that the bearded vultures stain their plumage to signal dominance status. The red color signal is only available at the cost of knowing or commanding a red ochre pool [10]. Such an explanation is relevant since cosmetic coloration occurs in 13 bird families and is considered to be a sexual signal [16]. Birds showing such activity invest much effort in plumage maintenance and improvement. For the latter purpose they use skin secretions, feather powder, or soil of different composition and color.

### 1.2. Outline of Research Hypothesis and Possible Alternative Concepts

**Figure 2:**
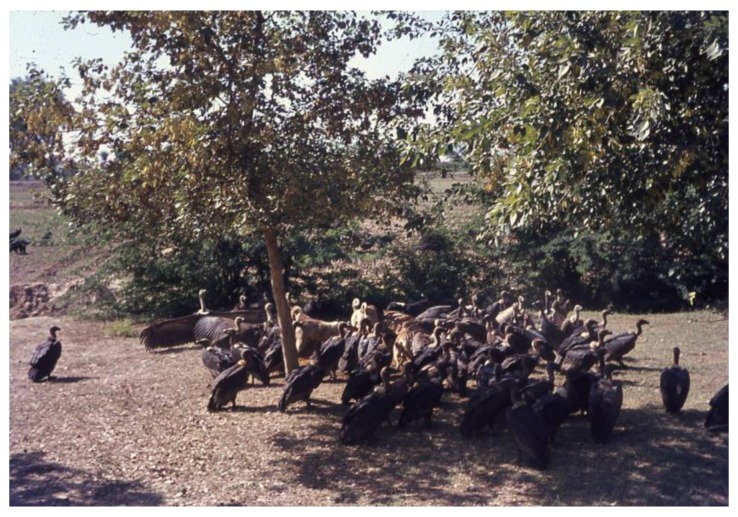
Carcasses of animals were in distant past intensively frequented by vultures. They must have been regular companions of men when feeding on slaughtered animals. (Picture from rural India, 1968, Helmut Tributsch).

In untouched environments, carcasses of animals are rapidly discovered and frequented by vultures. It is known that vultures play an important role for ecosystems in cleaning up potentially dangerous animal remains and avoiding the spreading of diseases [17]. More and more information is also available on the sensitive and balanced interaction between different vulture populations when using animal carcasses [18,19]. The crowding of vultures around animal carcasses is, for example, still observed in African national parks, where vultures are regularly competing with lions and hyenas for the dead prey. Four decades ago, vultures around carcasses were also a frequent picture along roads in India (Figure 2). Since then the vulture population has been decimated by environmental pollution.

Long ago, when primitive man was killing large size animals and recovering their usable parts, vultures became regular companions, competing for food. Primitive man is known to have broken bones for food. The bearded vulture also breaks and eats bones [4,5,6,7]. Whenever man was moving away from or leaving large carcasses of hunted animals, crowds of vultures were taking over. Man thus learned to know their habits. In Tibet and other central Asian countries, dead human corpses were even offered to vultures for a burial procedure. Primitive man must have observed that the bearded vulture, once a very common bird, after feeding on the carcasses, with the feather cover tainted with blood and other body liquids, regularly looked for red ochre pools for bathing.

Afterwards he apparently looked and felt much better. In an effort to get rid of the smelly remains on his body after cutting, treating and eating his prey man may have also tried to bathe in ochre mud. He found that this had a favorable medium term effect. The hypothesis advanced here, consequently, is that the ochre use by man had its origin as a bio-mimetic approach. Man was learning the procedure from a wild animal, which was competing with him for the carcasses of his kills. He found out that there were benefits. This publication will gather evidence for such a conclusion, study the reason for the attractiveness of red ochre, and examine the consequences to be drawn in anthropological context. It should be tested here, whether new knowledge and new inspiration can be deduced from a bio-mimetic approach to old anthropological questions. The aim is also to point attention to the benefits that primitive man has drawn from the skills and adaptations, which a vulture has acquired during evolution.

Anthropological science documents, that ochre has widely been used as a substance for decoration and ritual during human evolution [20,21,22]. This implies that the idea to use ochre came from the head and spirit of early people. The expected motives were: interest in decoration, ritual practices, mysticism and superstition. It is theoretically of course possible that ochre use by man developed independently from ochre use by the bearded vulture. Evolution has shown many remarkable cases of parallel evolution. However, later, arguments will be given which show that this is improbable. One is that Neanderthal people used ochre much earlier than modern man. This suggests that ochre was used by them as a tool for pure survival. How could its complex ritual use have been invented by such early and unsophisticated human beings? The other argument is that ochre was applied long before ritual practices became evident in human societies. For what other purpose was ochre used if not for decoration and rituals in context with symbolic behavior? Before answering this question, some information has to be given about red ochre.

Several types of natural red iron oxide pigments are known. They range from red earth, containing finely divided haematite (α-Fe_2_O_3_) mixed with clays and other accessory minerals, to relatively pure haematite ore. In more recent time yellow earth of iron sulphate was heated to produce red “burnt ochre”, which contained haematite. We will here identify ochre with earth mostly composed of red haematite. Up to now, interpretations on the background of the tradition of ochre use by ancient man focus on its decorative red color, which also resembles that of blood, a substance of life. It was assumed that painting the body with red ochre was a decorative symbol for life, survival and vitality. However, to the many authors who wrote about the use of ochre in anthropology [20,21], it was not known that ochre, when exposed to ambient and skin humidity, and to solar light, has also an additional property, which could have been relevant for human evolution. It is its property to produce, in presence of light, reactive chemical species, which can sterilize the skin and liberate it from odors. Such a property could have easily been discovered during evolution as the following imagined situations suggest.

Let us think of a band of prehistoric hunters in the northern tundra—plains of Eurasia. Following the example of the bearded vulture they have tainted their skin with red mud. When going on a hunting trip they easily make an important discovery: the annoying insects stay away. There is apparently nothing left in this red mud which is interesting for them.

Let us imagine another situation: a clan of migrating people of hunters and gatherers is hit by a strange contagious disease. Some people are dying, others going through an agony of pain. However, a strange phenomenon is observed. Those persons who had painted their bodies with red ochre and have red-stained hands, were not affected. The shaman realizes: the red ochre is a sacred material, a material which can heal and protect against the influence of “bad spirits”.

These two stories may not be fiction, but may reflect a reality, which our ancestors have lived, because ochre, exposed to daylight really does have self-cleaning and sterilization properties. Light produces radicals, reactive chemical species, which oxidize smelly organic compounds to neutral, volatile carbon dioxide, and which kill bacteria and viruses which populate dirty hands and the skin. It may have been these sterilizing, cleaning properties that have contributed to the attraction of ochre for rituals related to birth and death. It makes such critical situations safer, cleaner and free of otherwise abundant flies. This contribution aims at introducing the cleaning and smell preventing properties of red ochre into anthropological considerations and simultaneously at understanding its advantage for the bearded vulture.

### 1.3. Evidence of Ochre Use from Prehistory

What do we know about ochre use in prehistoric societies? Early evidence of ochre use by primitive man dates back 70,000 to 250,000 years. Recently it was demonstrated on the basis of excavations at Maastricht Belvedere in The Netherlands, that Neandertal people used ochre as early as 200,000–250,000 ago [23]. At this Neandertal excavation site, 15 small concentrates of iron oxide were found, believed to have originated as drops from an ochre-rich liquid substance. Since the nearest haematite source known is at 40 km from the site, the Neandertal people must have transported and used it. In Africa, red ochre became a common discovery in rock shelter sites from 160,000 years ago onward (cited in [23]). Red ochre stained bones were found in the Qafzeh cave in Israel, dating back 100,000 years. Seventy-one pieces of ochre were found placed along the bones of a buried person. They seem to have been used in a symbolic ritual. In the Blomos cave in South Africa, pieces of ochre were found together with 30 tools made of bones, dating to between 70,000 and 100,000 before our time. One of the ochre pieces showed cross hatched cuts, which apparently had some meaning. A 100,000 year old processing workshop for ochre at Blomos cave was identified [24]. Also a 38,000 years old skeleton found at Lake Mungo in New South Wales, Australia, bears traces of ceremonial ochre.

From the Aurignacien of Western Europe, 40,000 to 30,000 years ago, many burials are known which showed the work of caring people. This is evidenced by the fact that the dead or their surrounding were carefully arranged. In many cases, red ochre was distributed over the corpses, sometimes only over the heads. This can clearly be deduced from the coloring of the earth and the bones. One example is the burial of a man in the cave of Cavillon near Mentone at the Riviera on the Mediterranean sea. He was covered with red ochre. In the Barma-Grance cave only the head of a buried man was covered with ochre [25]. Other examples are the Cave of Cavillon, Liguria, and the Comb-Capell site in the Dordogne.

In Portugal, archaeologists found a complete skeleton of a young child dating back 28,000 years. It was covered with red ochre, and apparently buried with ceremony [26].

Twenty-six thousand years ago, in the Red Lady of Paviland Cave in Wales, Great Britain, a body was laid into a shallow grave and scattered with red ochre.

From a former hunting camp from the ice age, 25,000 years ago, in Russia, a burial of two children, 13–14 years old, is known, which is conserved in the Vladimir museum. They were bedded into ochre, which was mixed with approximately 6000 ivory pearls, which were originally apparently fixed to their clothes.

Another relevant grave, of a woman of mammoth hunters, is reported form Vestonice in the Czech Republic. She was put on her side, bound with a strap into a cover down position. She was decorated with teeth from polar fox, armed with stone weapons and provided with meat for food. She was strewn over with ochre and covered with two mammoth shoulder blades.

Also from 25,000 years ago a child burial from Portugal shows similar evidence of a ritual role of ochre. It comes from the cave Abrigo do Lugar Velho in the Lapedo valley. The body of the child was apparently entirely covered with ochre.

Approximately 13,000 years ago Cro-Magnon people from the Ofnet cave near Holheim in Bavaria arranged the heads of 4 men, 9 women and 20 children in two troughs. They were covered with ochre and asches [27].

From the North American Clovis culture, 12,000 to 11,000 years ago, ochre use for burial practices is also known.

During the middle and late Stone Age haematite or red ochre was used throughout South Africa. It is documented by red ochre stained grinders, its presence in burial on the bones, in hair or containers, on stones. In both Hottentot and Khoisan burials, ochre is documented for cosmetic and ritual use. Sometimes it is conserved in ostrich eggshells [28]. 

**Figure 3:**
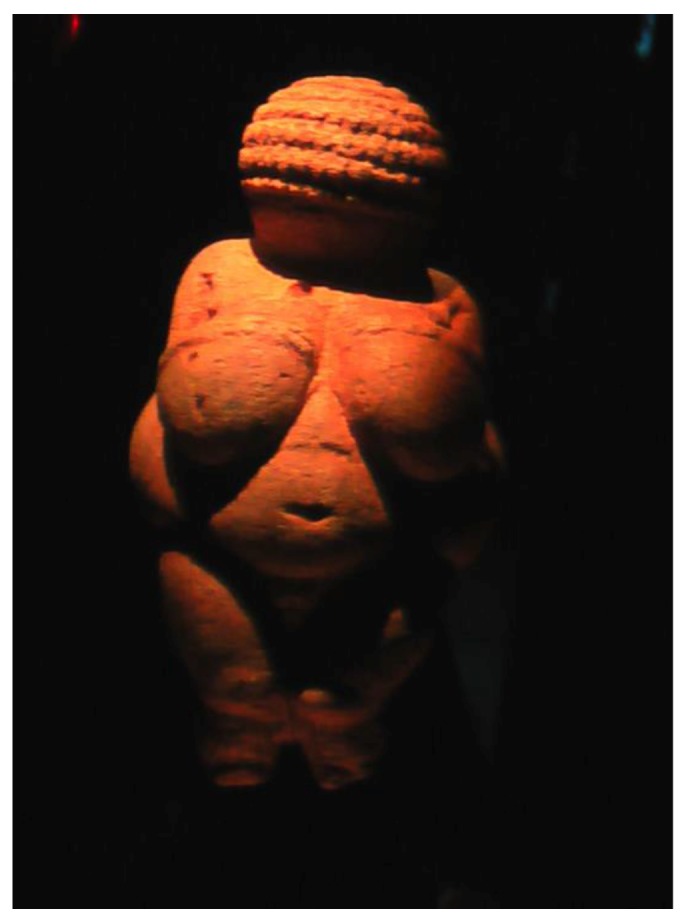
The Venus of Willendorf, a stone age symbol of fertility, estimated to be 24,000 to 22,000 years old, was once covered with red ochre (Natural History Museum, Vienna; photography by Helmut Tributsch).

From the older stone age in Austria small stone statuettes of opulent women are known. For example, a statuette from Mauern near Neuburg or another one known as the Venus of Willendorf (Figure 3). These obvious fertility symbols were both colored with red ochre.

Ochre was thus apparently not only used for burial, but also for birth rituals. In fact, red bed-clothes were customary in Germany up to the Middle Ages as a protection against the so-called “red illnesses”: These were, for example, fever, rashes, but also miscarriages. A famous document, which shows such a bed is the Arnolfini Wedding by Jan Van Eyck from 1434. It may have been based on a subconscious memory on red ochre being good for health and birth. Red wedding gowns were, by the way, still used in Nürnberg, Germany, during the 18th century following a widely spread tradition of red gowns and veils that can be traced back to Roman time and Chinese culture. A protective power against evil spirits was attributed to the red color.

However, ochre also was used in combination with animal sacrifices and to conjure the fertility of animals. In many paleolithic caves, ochre, in combination with fat, was used to paint the first symbols, which man invented: figures and lines of red dots, geometrical patterns, circles, impressions of the human hand. The walls of megalith stone graves also showed ochre paintings, for example the megalith grave from Leuna-Gölitzsch near Merseburg in Germany.

**Figure 4:**
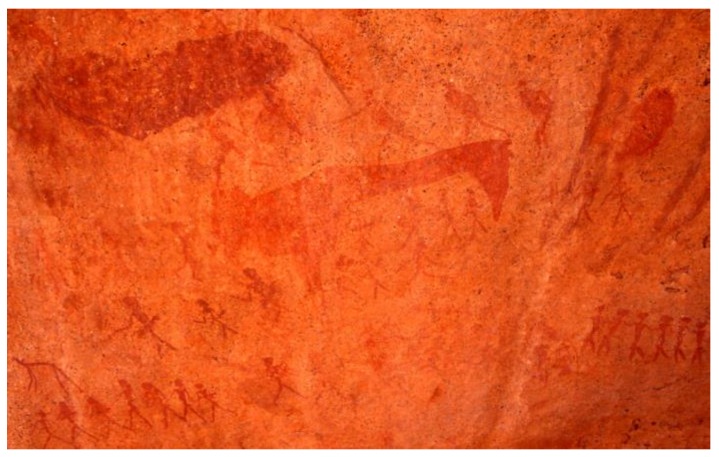
Ochre bushmen rock painings from Brandberg, Namibia. The bushmen mixed red ochre with blood or egg white to paint social and ritual activities, or the mythical rain bringing giraffe as seen here (Helmut Tributsch).

The ritual or protective use of ochre continued into our time. The bushmen sometimes anointed the dead bodies with red powdered ochre and melted fat. However, they also liked to paint ritual ceremonies on rocks with red ochre (Figure 4). Bushmen in Botswana used a pomade of mud and dung with ochre for their hair. Ochre was also widely used by Bushmen as pigment for cosmetic purposes. Among Hottentots, red ochre mixed with fat was used as a ritual paint and body paint. The same was reported for the aboriginals of Tasmania. The Nama tribe in Southwestern Africa still uses red ochre.

**Figure 5:**
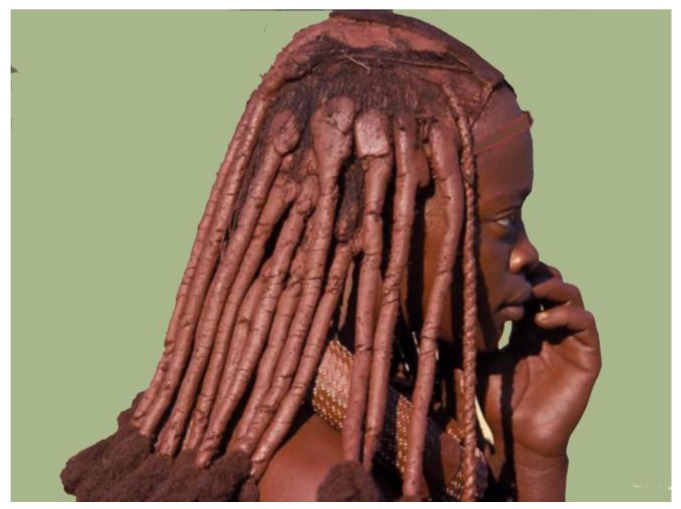
Himba women use ochre mixed with fat and herbs to treat daily the skin and the hair. They consider this as a cosmetic as well as health-supporting procedure (Helmut Tributsch).

Also for the Himba tribe in northern Namibia ochre is still part of their tradition. Women and older men use ochre-fat on a daily basis to take care of their skin, instead of washing it with scarce water (Figure 5). Two cosmetic boxes made from wood or horn are used by the women. Ochre powder mixed with aromatic herbs and bark is kept inside one, and fat inside the other. The substances are mixed and rubbed onto the body. Also when boys of the Massai in Kenia grow up, their heads are shaved and ochre with fat is rubbed into the skin.

All these examples show that ochre has persistently accompanied prehistoric man through everyday life, including birth and death. The use of red ochre in burial practices of North American Algonquin people 3800 to 3400 years ago was so characteristic, that their culture was even named Red Ochre Culture. The same is true for the Yamna culture from the late copper/early bronze age in the Bug/Dniestr/Ural region of Russia (3600–2300 BC), which was also called Ochre Grave Culture, because inhumations in kurgans (tumuli) involved covering the bodies with ochre. Was it just the red color that made this powdery paste so attractive as present interpretations assume? Did the bearded vulture only strive for red-brown beauty to signal dominance status? Or was there a property behind ochre treatments, which is favorable for health and the quality of life? Modern science, by better understanding the properties of red ochre, can shed new light on the ancient fascination with this natural substance.

## 2. Results and Evidence for a Practical Use of Ochre

### 2.1. The Photo-Fenton Reaction and Photo-Catalysis Pathways with Ochre

It has been known for a longer time that iron containing solutions and suspensions containing organic molecular species are photoactive in generating aggressive or radical species such as OH**^•^**, O_2_^−^, HO_2_, H_2_O_2_ during illumination with visible light. Especially the OH**^•^** radical is very efficient in oxidizing organic molecules to volatile carbon dioxide [29,30,31,32,33,34,35]. In all cases studied, the iron species is involved, either as the Fe^2+/3+^ redox species, as iron in the form of diverse organic Fe^3+^ complexes such as, for example Fe^3+^-oxalate or carboxylate, in the form of Fe^3+^ hydroxo-complexes or in the form of iron oxide (haematite, α-Fe_2_O_3_) particles, in which iron is present in the form of Fe^3+^ [36]. While the detailed mechanisms are complex and may somewhat differ in different molecular and mineral environments, they may be generalized under the term “Fenton”-reaction, since Fenton described the role of iron species in these reactions in combination with hydrogen peroxide. Since many soils have a high content of iron oxides and hydroxides, as well as of humic and carboxylic acids, these photo-generated mechanisms of radical formation involving iron species play a significant role for environmental and aquatic science [37]. In analogy to mechanisms in natural environments, iron based photochemistry and radical formation has been developed for wastewater treatment and tested at the Plataforma Solar de Almeria, Spain [38,39].

The key process involved in these systems is an efficient electron transfer from ligands or organic species to the ground state of solar excited Fe^3+^, to form a Fe^2+^ species, which then donates the surplus electron in the energetically high positioned excitation level. Oxygen can now be reduced to O_2_^−^ and further to H_2_O_2_. It may then be further reduced, thereby forming OH**^•^** radicals and OH^−^. The interplay of light induced hydrogen peroxide formation form oxygen and its reductive dissociation into radicals generates a chemical environment which is aggressively oxidizing and sterilizing for micro-organisms. Many reaction rate constants or equilibrium constants of the responsible mechanisms of iron species have been measured and compiled [40].

**Figure 6:**
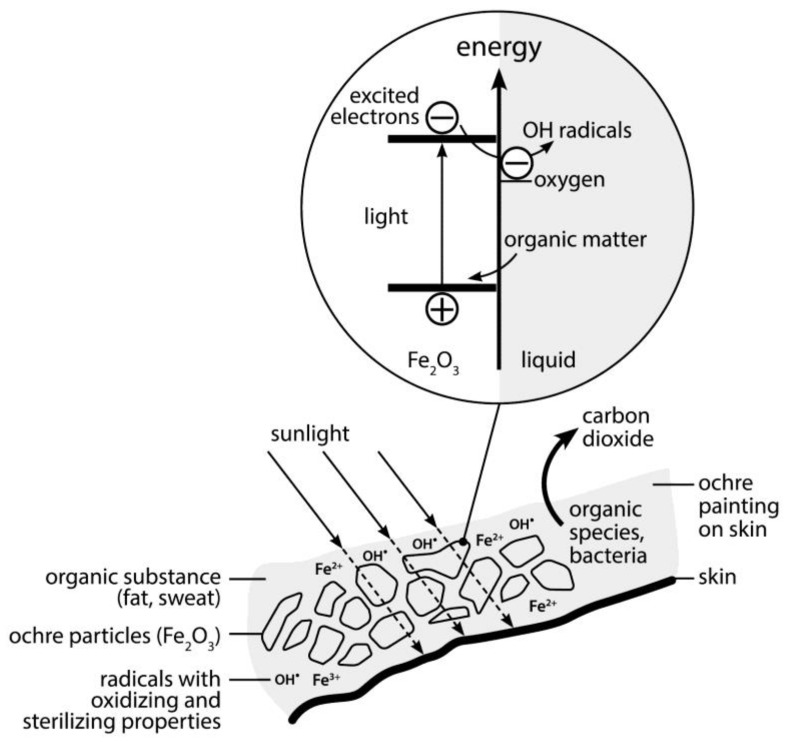
Scheme explaining the action of ochre (mixed with organic substances like fat) on the skin. In the outer layer visible light (with energies exceeding 2 eV) is absorbed. Electrons from the organic components are filling the empty electronic ground state and suppressing recombination of the excited electrons, which are then reducing oxygen to form peroxide and OH radicals. They eliminate smell and kill bacteria and viruses.

As already mentioned, dispersed haematite particles are also critically involved in the described iron mediated photochemistry leading to the formation of OH**^•^** radicals. Commercial grade haematite (α-Fe_2_O_3_) has been used to study the effect of illumination of haematite suspensions on pathogenic bacteria such as *Escherichia coli*, *Salmonella typhi* and *Shigella flexneri* [41]. The bacteria in concentrations of 10^4^ cells per ml exposed to the light of a 250 W tungsten lamp were killed within 30 min (the number of viable cells was checked by cultivating cells after suitable dilution on Nutrient Agar Medium). The photocatalytic effect of radical formation with α-Fe_2_O_3_ with respect to sterilization of bacterial cultures is thus comparable to the effect known for illuminated TiO_2_ which absorbs 3% of solar light in the UV and is now already commercially used as a substrate for sterilization of surfaces, for example of ceramic tiles and surfaces in hospitals. It is also used for self-cleaning facades and surfaces, which take advantage of UV-generated radicals, that transform organic dirt into volatile carbon dioxide [42]. Illuminated α-Fe_2_O_3_ has qualitatively similar radical producing properties like TiO_2_ and the additional advantage of absorbing visible light with an energy as low as 2 eV (Figure 6). This is the optical transition between the energy bands in haematite, which transfers electrons from the lower placed valence band into the higher conduction band, which is responsible for the red color of this mineral. It allows a subsequent electron transfer from a donor molecule to the valence band and results in a following up transfer of the originally excited electron to molecular oxygen leading to hydrogen peroxide formation. OH**^•^** radicals are thus formed under illumination. They deteriorate all kinds of chemicals including pesticides [36]. A disadvantage of haematite with respect to technical applications, compared with the radical generating, self cleaning TiO_2_ is its inferior chemical stability. However, long term stability is not a problem with an ochre layer on the skin. TiO_2_ is very rare on earth. Titanium typically occurs in the form of Ilmenite, FeTiO_3_, which does not have photo-catalytic properties. Ochre is therefore the only abundant mineral on earth we presently know to develop photo-catalytic radical generating activity. Only the rare UV—light absorbing ZnO, with its white color derived from light scattering, has comparable properties. However, ochre is abundant on earth and was therefore accidentally discovered with respect to its favorable properties by the bearded eagle and later early man and subsequently used all over the world. The ochre, mixed with organic compounds and exposed to the skin will photo-react in sunlight and may gradually alter its chemical composition with time. The iron species, which is crucial for the radical (OH**^•^**) forming photo-Fenton reaction, remains in the system and works, regardless whether the Fe^3+^ species is part of the crystallized micro- or nano-crystalline haematite layer or part of a dissolved organic iron complex in a moist layer of sweat on the skin.

### 2.2. Solar-Sterilizing Ochre and Man

Fact is that ochre mixed with fat or other organic substances in combination with the sweat of a sunlight exposed human skin provides a favorable production and reaction medium for aggressive OH**^•^** radicals. They kill bacteria and oxidize organic compounds gradually to carbon dioxide, which is volatile. An ochre treated skin is thus expected to be gradually purified in the sun, both from smelly chemicals and bacteria and other micro-organisms. Flies will stay away. In addition it is protected against excessive radiation, because ochre is filtering out the high energy portion of visible solar light and also its UV component. A person painted with ochre and carrying ochre color on his hands will also have a much smaller chance to transmit contagious diseases when in contact with people and will also protect herself. Ochre is thus associable with health and protection, but also with cleanliness, since smelly components of sweat will be oxidized to carbon dioxide. In an environment with abundant flies, such an effect may readily be felt.

We cannot exactly reconstruct how prehistoric people have used ochre when confronting diseases and cosmetic problems. However, archaeological evidence clearly shows that the properties of ochre were appreciated in critical situations such as birth and death. Painting fertility goddesses with ochre (Figure 3) may have meant that ochre was typically used during birth and was considered essential for a positive outcome of birth. Smearing ochre onto the body of a highly pregnant woman and her midwife for a birth ritual may have reduced the risk of childbed fever. The ochre on the skin exposed to daylight may have gradually sterilized it, and would also have kept off flies. It was mentioned above how effectively illuminated ochre can, for example, kill *Echerichia coli* bacteria.

Covering dead corpses with ochre may have meant that ochre should provide comfort for the soul on her way, should avoid contagion for the mourning relatives in the case of a disease, or it should have simply avoided bad smell and flies. It may be that people were treated with ochre also during their agony before dying, in the hope that death can be avoided. Treating a dead body with ochre may not only have been a spiritual symbol, but was simultaneously a precaution against flies and disease, and thus evil spirits in the eyes of prehistoric people.

This interpretation of the prehistoric significance of ochre may be seen in context with other ancient rituals the original meaning of which has been forgotten with time. An example is incense burning, which has been applied in ceremonies of numerous religions since antiquity. Modern science could show that the smoke of incense is an aggressive sterilizing agent including components that are carcinogenic and thus even harmful for people at the long term. The smoke particles, depositing on the interior surfaces of temples and churches make them deadly for micro-organisms. The interpretation is that burning of incense and distributing the aggressive smoke was adopted to reduce the danger of contagion of disease during the necessary gathering of people during religious ceremonies. Today we may ask how this purifying effect of incense burning was discovered by early priests or shamans.

Ochre may have played a similar role in protecting people.

### 2.3. Traditional Explanations for Ochre Use

Previous interpretations of ochre use involve different explanations. In the case of the ochre painting of the Venus of Willendorf (24,000–22,000 BCE), which was identified at the time of discovery, the theory was advanced that the red ochre paint served as a surrogate of menstrual blood [43,44]. This would place the figurine within the sphere of the female. The artist may have been a woman.

The use of ochre in burial rituals has also been intensively discussed. It has been suggested that early humans began to paint their body with ochre as a kind of signal, to communicate a message to other humans [45]. Since ochre stained tools have been found in cave burials, it was also speculated that ochre production was probably part of the burial ritual. It may have expressed a symbolic concept. It has also been proposed that ochre may have simply symbolized death [46]. It has, as mentioned above, also been suggested that red ochre may have been a symbol of menstrual bleeding. Women may also have painted themselves red to pretend fertility or to confuse menstrual signals [35].

These classical interpretations of ochre use in human prehistory and history, which cannot explain the fascination of the bearded vulture for ochre tainting, should now be re-examined in the light of the proposed life protecting sterilizing and smell preventing properties described for ochre. 

### 2.4. Evidence for Ochre Utility from Beothuk—Red Indians and Himba

It is a remarkable fact that ochre use has continued to our time (see Figure 5) so that historic documents and contemporary studies can partially be used to verify the presented hypothesis, that it was favorable for health and prevented smell. Some aboriginal people in Australia still carry on a 50,000 year old uninterrupted tradition of ochre use. After fabricating a spear with great care they regularly rub the shaft with kangaroo blood. The superstition is, that the spear will henceforth be drawn towards kangaroos. However, together with the kangaroo blood the aboriginal hunters applied ochre to the spear shaft [47]. This means, that here ochre is not a substitution for blood, as some scientists believe, but has a complementary function. Who would like to touch blood with the bare hand, especially when imagining its attraction for insects and its deterioration? Via its association with blood, ochre transforms the tainted spear shaft into a tool more pleasant to deal with. It appears to be the sterilizing, cleaning property of ochre, in combination with a biological liquid, which is of interest here. In aboriginal Australia, a father of a new born child used to take the child’s umbilical cord, to paint it with red ochre, and to send it to distant relatives as irrefutable evidence of birth [47].

From the coast of Labrador we know of a 7500 year old burial of a 12 year old boy, wrapped in furs and birch-bark and dusted with red ochre. When the Norsemen arrived in the same area 1000 years ago and when John Cabot revisited the site 500 years ago (in 1497) ochre painting was still in use. The Beothuk Indians used to literally cover their entire body with red pigment of ochre. They applied it as a cosmetic as well as to paint tools, weapons and clothing. It is documented that the Beothuk believed that death was a form of sleeping and that red ochre had “life giving power”. This report on the spiritual meaning of ochre is very relevant for our interpretations.

In the Beothuk burial ceremony, red ochre was used and the deceased and his belongings were painted red. It is also reported that the Beothuk Indians estimated red ochre as an effective remedy against insect bites. It is interesting to note that the ochre red appearance of the Beothuk tribe conduced to the name “Red Indians” given to all American tribes.

Most of the other North American tribes used ochre to some extent. However, they could apparently not always distinguish between red ochre and occasionally found rare mineral earth of identical color but different composition. This can be derived from the fact that Indians in Mexico today occasionally use red cinnabar, mercury sulfide, of volcanic origin, which they apply to their votive statues in request for benefits. This reminds of the ochre stained Venus statues from paleolithic time (Figure 3), but this substance is toxic. It may have locally replaced red ochre just because it was mistaken for this mineral or because the red color has become the dominating feature. Also from the 8000 year old archaeological site of Hüyük in Anatolia, a cinnabar red stained skull is known. The red mineral was obtained from the nearby volcano Hasan Dag and apparently mistaken for red ochre.

A tribe which continued the use of ochre in Namibia, Africa, are the Himba (Figure 5). Their women traditionally paint their entire body with ochre including their hair, clothes and tools. Instead of washing themselves with (scarce) water in their desert environment they cover their body in the morning with fresh ochre, animal fat and herbs in a body massage. Himba appear to use the ochre treatment for cosmetic reasons. It keeps them from smelling bad and protects them against the intense African sun.

Also, the vanishing Onge tribe from the Little Andaman island used ochre painting of the face. It is reported that they apply ochre painting both against insects and for decoration.

This modern information on ochre use appears to support the interpretation of the benefits of this remarkable substance presented here: It is life-supporting. It protects against insects, bad smell and sun burn. It was assumed to provide life giving powers and it was apparently for this reason why a deceased person and her possessions were covered with it. Ochre should help to overcome death and to start a new life in the other world. It was also expected to help starting a new life in this world. When an aboriginal man in Australia was killed for an offence via the secrete Mulunguwa procedure in the Alawa tribe, his widow was subsequently washed in a hole by other women and painted with red ochre. This freed her from her husband’s shade [47].

Ochre was expected to have the power to fend off or to overcome death bringing disease. Is it, on the basis of such credits, surprising that the origin of man is associated with ochre? The name of Adam, the first man, means nothing else than “red earth”. It means that ochre was expected to promote life, that it even helped in the creation of the first man. In spite of its alarming color, resembling the color of blood, in some cultures it is also identified with beauty. People are attracted by it. The “Red square” in Moscow means beautiful square.

## 3. Discussion

### 3.1. Ochre Benefits for Survival

In prehistoric times, epidemics of infectious diseases in humans are considered to have been comparatively rare. The hunter-gatherer communities lived as small migrating bands of people with little contact to other populations. Their changing environment during migration remained clean and there were no domestic animals to transmit diseases. The problems became much more serious with the arrival of animal breeding and with agriculture, which saw the crowding of people in increasingly large settlements [48,49,50,51]. New challenges have later arrived with environmental pollution, overpopulation, degradation of the environment, mass tourism and deteriorating of water resources.

However, let us reconsider the danger of contracting diseases in prehistoric hunter-gatherer societies closer. Men were regularly hunting, cutting into pieces and transporting all kind of wild animals. Occasionally they were dealing with sick animals and, in case of hunger, with half rotten carcasses. Women were caring for the preparation of skins and furs as well as for the recovery, for technical use, of animal parts such as intestines, bladders and tendons. During such activities, which were essential for sustaining prehistoric life, people came in intensive contact with fleas, lice, bugs and all kind of parasites which are present in furs, feather skins and organic matter. They can transmit various severe diseases. This is also true for intestines and excretions of animals, which may carry all kinds of parasites. Prehistoric people may not have had the practice of regularly washing their hands, because they did not have any knowledge about the spreading of diseases. They also did not know that pathogenic bacteria can rapidly develop on high-protein food such as meat, poultry, fish and eggs, which were highly estimated by hunter gatherer groups and carried along during migration. Among the commonly found pathogenic bacteria (besides the majority of harmless ones) one should mention *Salmonella*, spp., *Staphylococcus aureus*, *Clostridium perfringens*, *Listeria monocytogenes*, *Campylobacter jejuni*, *Vibrio parahaemolyticus*, *Bacillus cereus* and *Escherichia coli*, in particular *E. coli* O157:H7. *Salmonellae* are present in the intestinal tracts of humans and animals. They may easily be transmitted by undressed people sitting together in temporary shelters. The pathogenic food poisoning bacterium *Staphylococcus aureus*, for example, lives today in the bodies of half the human population. Infected migrating animals may occasionally have posed an additional hazard, as seen with bird flue in recent times.

It may be asked whether red ochre has also been used to kill bacteria and fungi for preservation of food. Indeed, there is an example from the north coast of Australia. The aboriginals, who otherwise preserved very little food, did this for preserving plums. They dried the plums in the sun, then rubbed them with red ochre, and dried them again [52].

A further significant problem for prehistoric man were insects which made certain regions practically uninhabitable because they were transmitting diseases like Malaria and Dengue fever. Ochre painting of the body and tools may have helped prehistoric people unknowingly to keep insect pests off and to enter insect infested regions more safely until they developed a certain degree of immunity.

In intensive sunshine, the concentration of bacteria is typically reduced. A strongly illuminated ground is sterilized to some degree through UV-A and -B radiation. This radiation damages the skin of man. Red ochre on the skin would also shield off dangerous UV radiation and simultaneously absorb visible sunlight to generate sterilizing and oxidizing radicals. The bacterial fauna contaminating the skin would be destroyed, and would not find a suitable substrate for development.

Red ochre on their skin in sunlight may have purified the body surface from ill-smelling organic substances, which are photo-oxidized to volatile, neutral carbon dioxide. This may also have prevented parasitic organisms from smelling human hosts. The same may be true for certain insects such as the Tsetse fly which is responsible for the transmission of Malaria. This hypothesis could, by the way, in the future be experimentally tested both in the laboratory and with native tribes, which are still using red ochre for skin protection. An additional favorable property of ochre is its strong optical absorption of light from the ultra-violet region (UV-A, UV-B) and the shorter wavelength visible region. It consequently prevents the dangerous UV light from reaching the skin and keeps off the blue and green portion of the sunlight, which is most intensive and still harmful in presence of sensitizing molecules. It is known that excessive sunburn deteriorates the immune system of the body. An ochre painted skin may have helped to protect the covered body while generating a sterilizing layer at its outer surface where light is strongly absorbed and produces carbon dioxide generating radicals (Figure 6). The knowledge of the beneficial, infection avoiding action of red ochre on the skin appears to have survived until the Greek civilization recorded it. The warriors of ancient Greece used to paint their body with red ochre, before going into battle. This procedure was expected to help wounds to heal better. In fact, medicine men from aboriginal Australia had a cure for yaws, a disease mostly known from poor native communities. This is a chronic infection, which affects mainly the skin, bone and cartilage and is caused by the bacterium *Treponema pertenue*. It starts at a single infected lesion and develops lesions all over the body. The aboriginals applied a poultice of wet red ochre to cure it. Anyway it kept also flies away from the body [47].

Now that chemical science has explored the OH**^•^** radical producing property of ochre, science of prehistory may critically analyze the here presented considerations suggesting that prehistoric man has long ago discovered its benefits and incorporated them into medical, hunting and magic practices. Red ochre is the only abundant mineral, which has the property of self-cleaning and sterilizing under sunshine conditions. It has helped man to conquer new habitats, to protect himself against excessive radiation, and to fend off insect bite and diseases.

What is good for the skin of a person can also be good for the surface of a house. In Scandinavia, Newfoundland and Labrador ochre is mixed with fish or vegetable oil and used as characteristic red-brown house paint. Wood-decay fungi may thus be suppressed. Also, traditional sails for sailboats were stained with ochre for long-term durability and Australian aborigines used it to preserve animal skins. The ochre layer itself is very stable. This can also be deduced from ochre paintings on rocks (Figure 4) which are also known to be very durable.

### 3.2. Smell and Human Survival

Today, we can no longer imagine the importance that smell could once have played in prehistoric societies in areas like social contacts, in hunting, in warfare and in protection against aggressive wild animals. Smell is, for example, also important for the contact and communication between people, especially between men and women during the fertile period of a woman. An ochre stained skin will convert organic molecules into odorless carbon dioxide. By using ochre, women could better hide their fertility to control the sexual behavior of men and could easier handle cleanliness problems. Moreover, most importantly, an odorless skin, and odorless weapons, were, on the other hand, also a significant advantage for a primitive hunter. He had once to approach wild animals very closely to make a kill. The extraordinary sense of smell of the prey has always been a critical barrier for success. In spite of his reddish appearance an ochre covered hunter became a more efficient hunter. Animals could not smell him. Having become a largely odorless hunter may have contributed to the success of man during evolution. It is known now that the availability of abundant protein food has been a decisive factor for evolution of the highly energy consuming human brain. Ochre covered hunters, following herds of wild animals, that frequently changed their direction against the wind, could have executed man’s domination of the animal kingdom. Controlling human smell may have been an important, and until now unrecognized factor in human evolution during his quest for more meat from hunting.

What evidence can we still find today in support of the smell eliminating function of red ochre? We can, for example, get it from the life accounts of aboriginal people [47]. In the iron oxide-rich environment of the Australian landscape, aboriginal hunters covered their skin intentionally with brown mud in order to get rid of their smell. Especially when they were very hungry, they regularly painted themselves with ochre for hunting in order not to miss the prey. They were so aware of the significance of smell, that, when approaching prey, they even tried to kill flies on their body. These flies were suspected to move to the prey at close distance, thereby warning it by carrying the smell of the hunter. Since suppression of smell for the hunter was so important, it is therefore not surprising that, in areas where ochre was not readily available, it was traded over large distances, often over hundreds of kilometers [47]. It was a strategic material, crucial for survival. It may have been equally relevant for inter-tribal warfare, since to well-trained noses smell could indicate the neighborhood of enemies, especially at night.

The until now unrecognized possible role discussed here of photoactive properties of ochre body and surface painting during evolution of man, its benefits for health, community life, hunting and survival, could in the future further be examined by straightforward experimental strategies. In my opinion, the properties of red ochre discussed here thus appear to more easily explain its outstanding role in life and death rituals during a period exceeding 100,000 years of human evolution than earlier discussed concepts, which may have played an inferior role. We will probably never with certainty find out exactly how the bearded vulture, *Gypaetus barbatus*, (Figure 1) himself discovered ochre bathing and how it developed into a genetically anchored habit. My understanding is, that the vulture occasionally mistook red ochre for ponds of blood. Searching in them for bones and meat he wetted his plumage with ochre mud. So he learned gradually to distinguish and found out the advantages of ochre treatment for his plumage. Application of sticky mud seems to be a contradiction to the delicate feather cover of a bird. However, rotting fluids from carcasses are not easy to handle. Moreover, a bird free of nasty flies and parasites may have been more attractive for mating and may have remained much more healthy.

The mostly bone eating bird must have developed the habit of ochre bathing long before the rise of man. With the assumption of a bio-mimetic learning of ochre bathing by early man and his discovery of the discussed advantages, the question of ochre use in prehistory has conducted us to an entirely new scientific interpretation. Since ochre use has been confirmed for South African human populations as early as 100,000 years ago [36], and for the distinct human branch of Neandertals in Europe as early as 200,000 to 250,000 years ago [23], there has possibly been an independent, parallel evolution. Since the bearded vulture is still roaming in both geographical regions, a parallel bio-mimetic learning from the bird’s habit was possible. The bearded vulture does not roam in America and Australia, where aboriginal people also used ochre painting. This is not seen as a contradiction to the presented hypothesis, since man has reached these continents only approximately 15,000 and 50,000 years ago respectively. He may have already known the benefits of ochre painting. The fact that already early Neandertal people have used ochre may support the practical significance of ochre bathing discussed here as compared with a spiritual, magic or aesthetic significance, attributed to it in traditional anthropological research. If the Neandertal population, 200,000 years ago, which is considered to have been intellectually much less flexible than modern man, already used red ochre, this supports the bio-mimetic interpretation advanced here. The early Neandertal man will have used red ochre for a practical purpose such as smell prevention, insect repelling or health, but not for ritual aims in birth and death ceremonies. For a purely symbolic use of ochre, bands of Neandertal people 200,000 to 250,000 years ago were, according to our present understanding, not sufficiently developed spiritually and intellectually. However, they were sufficiently evolved to copy ochre bathing from a vulture and to find out that they feel better afterwards. The evidence, from a Neandertal camp site, that an ochre concentrated liquid was used there [23], supports the conclusion, that the body surface was treated for a challenging hunting expedition, exactly as Australian aboriginals still did two hundred thousand years later [47]. For many years to follow the body comfort and the lack of smell may have motivated a continuous use of ochre. This could explain the persistent application of ochre in early human societies before symbolic uses in rituals were added because of the benefits of ochre. Such circumstances are also much more probable than a “discovery” of the beneficial smell avoiding and disinfecting activity of ochre on the skin by early man himself. This would have required a nearly scientific course of action. In addition the spiritually less developed Neandertal people in Europe and early modern man in South Africa would have had to discover ochre benefits independently, without learning it from the bearded vulture, who regularly shared his kills. 

### 3.3. Bionic Applications in Medicine

There are challenging problems in medicine, where the combination of skin or interface, ochre and light may offer new opportunities. This could be the problem of eliminating antibiotics resistant bacteria and other pathogens in hospitals, where the creation of clean environments and body surfaces is a very crucial issue. Iron oxide, ochre, is entirely harmless to human life. There are no side phenomena known and iron in a certain concentration is even needed in the body for health. This is a significant advantage compared to photo-reactive dyes, which can also generate radicals, but may decompose into unstable products, which could be damaging for health. Ochre requires only visible light between 400 and 620 nm for becoming photo-chemically active. UV light can thus be excluded. Visible light can now be conveniently, in an elegant way, and economically provided via LED technology. Also, safe chemical-reducing compounds, which may transfer electrons into the valence band of illuminated iron oxide are available. In hospitals, body surfaces, textiles, gloves and instruments, floors and walls should be experimentally treated with ochre under visible light illumination to fight resistant bacteria. The strategy, which was so useful for human evolution, should be developed to a modern standard on the basis of such a sustainable and health-compatible material.

## 4. Conclusions

### The Bearded Vulture and Modern Man

What early man has learned from the bearded vulture, to use red ochre on his skin, may have safeguarded him through dangerous and challenging periods of evolution. One should think of strenuous hunting expeditions, the handling of huge animal carcasses, daylong feasts, many flies and the danger of contagious diseases. Red ochre paining may have helped in all these situations. However, man has long forgotten that he may owe the secret weapon against smell, disease and also sunburn to the bearded vulture. For thousands of years the bearded vulture did not much interfere with the interests of man. However, modern times with their highly effective weapons, uninformed hunters, and toxic chemicals became a disaster for this proud bird. Fifty years ago, only 40 to 60 breeding pairs of bearded vultures were left on the European continent, mostly in the Pyrenees, on Corsica and Crete. Fortunately a conservation program started and in the Alps alone 128 free born bearded vultures are now circling again. Man should look at these elegant and skillful birds with special care and interest. They seem to be very independent and unrelated to man’s fate, but with their addiction to ochre bathing they have helped us to survive. We should be grateful for that and secure them a protected place in our modernized world.

This analysis shows that a bio-mimetic research strategy, a strategy in which man is expected to have learned from living nature, can contribute to the advancement of understanding of human evolution. It points attention to new facts and permits new questions and conclusions. It also shows how primitive man has taken advantage from a habit of an animal, a habit, which became a key element towards improved hunting, prevention of disease and evolution of rituals aimed at making contact to the supposed other world during birth and death ceremonies. By experimenting with an animal habit, man may also have been inspired towards spiritual frontiers and maybe also towards art, because the presence of red ochre in his environment, red ochre on his hands, may have motivated him to draw ochre paintings on rocks. Maybe he was spraying liquid ochre with his mouth over his hands to protect them against smell and insects, when he discovered the characteristic patterns on the rock surface below. The combination of fat with red ochre on his body may also have shown him the strategy to make such rock paintings last for thousands of years.

Such studies, bases on bio-mimetic considerations, also lead to a more profound understanding of animal behavior itself: the bathing of bearded vultures in red mud with the subsequent exposure to the sun can now be better understood. It has the purpose of getting rid of parasites, microbes and smell from the feather coat. The ochre treatment of the feather coat could, of course, later also have evolved into a signal of dominance status [10]: it would be a signal of cleanliness and health.
